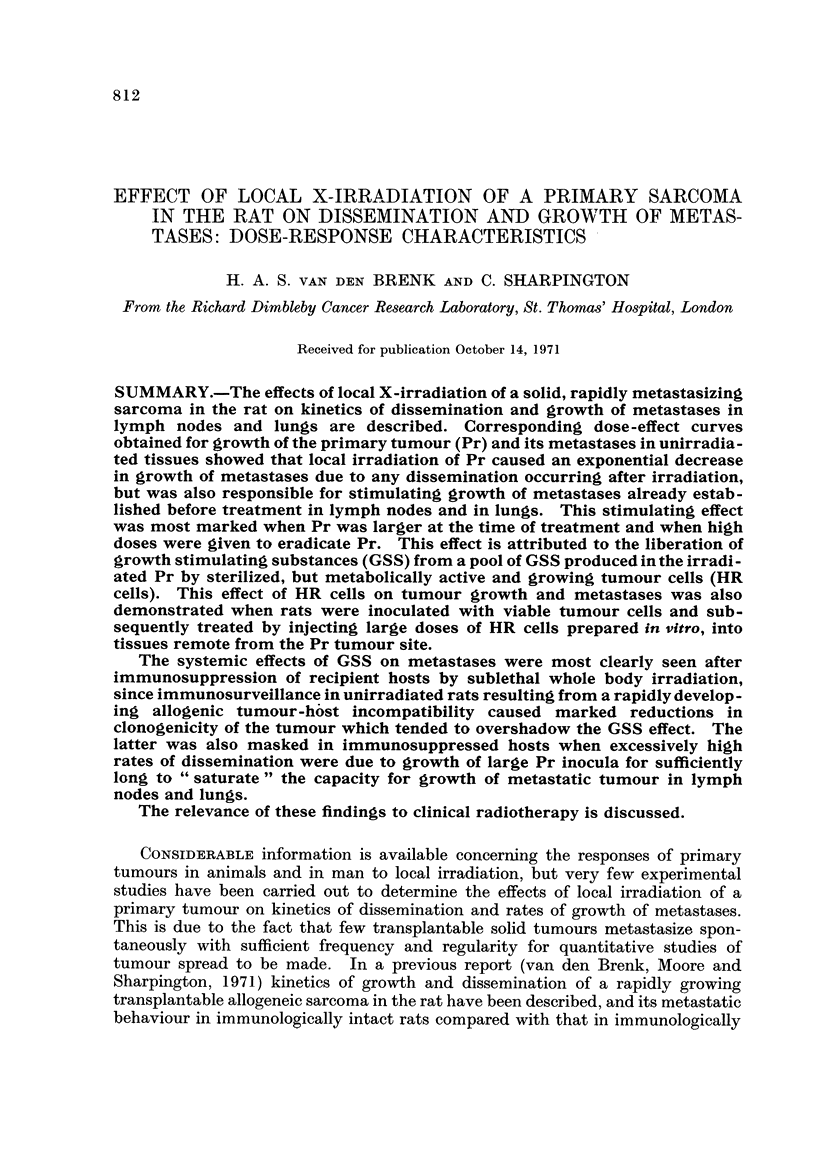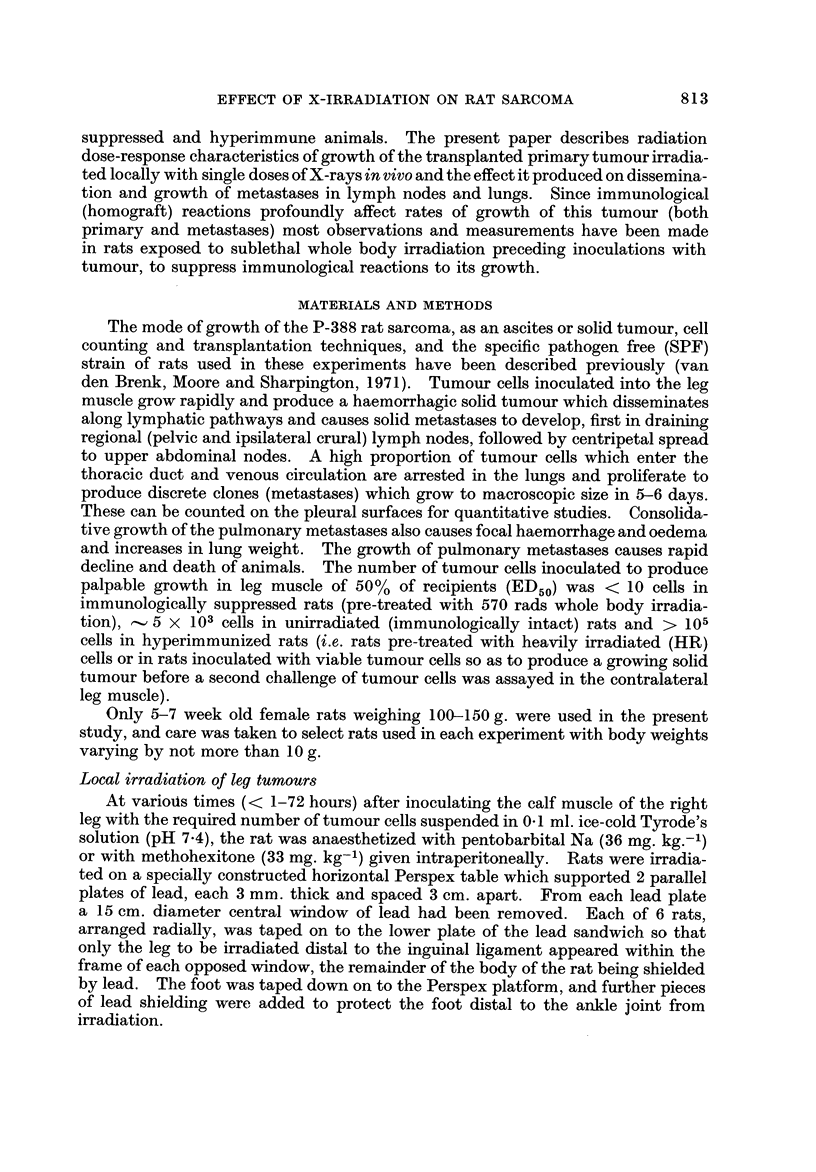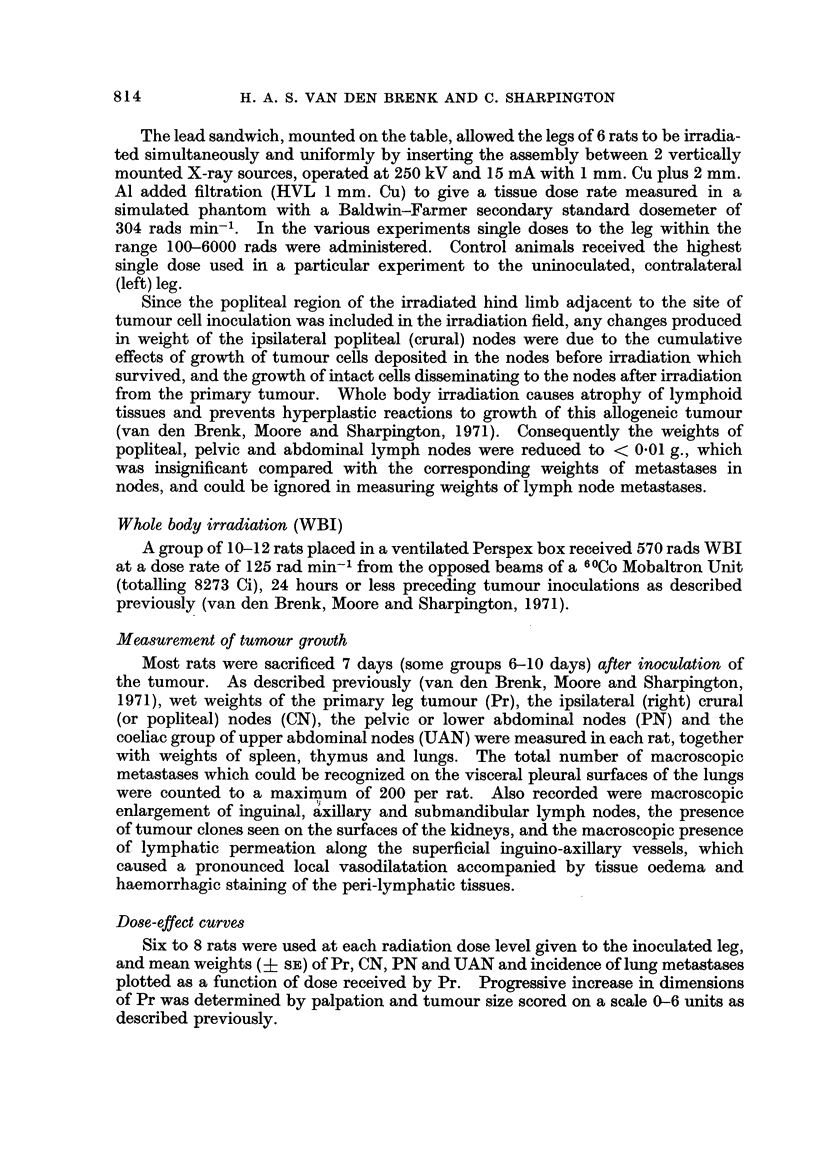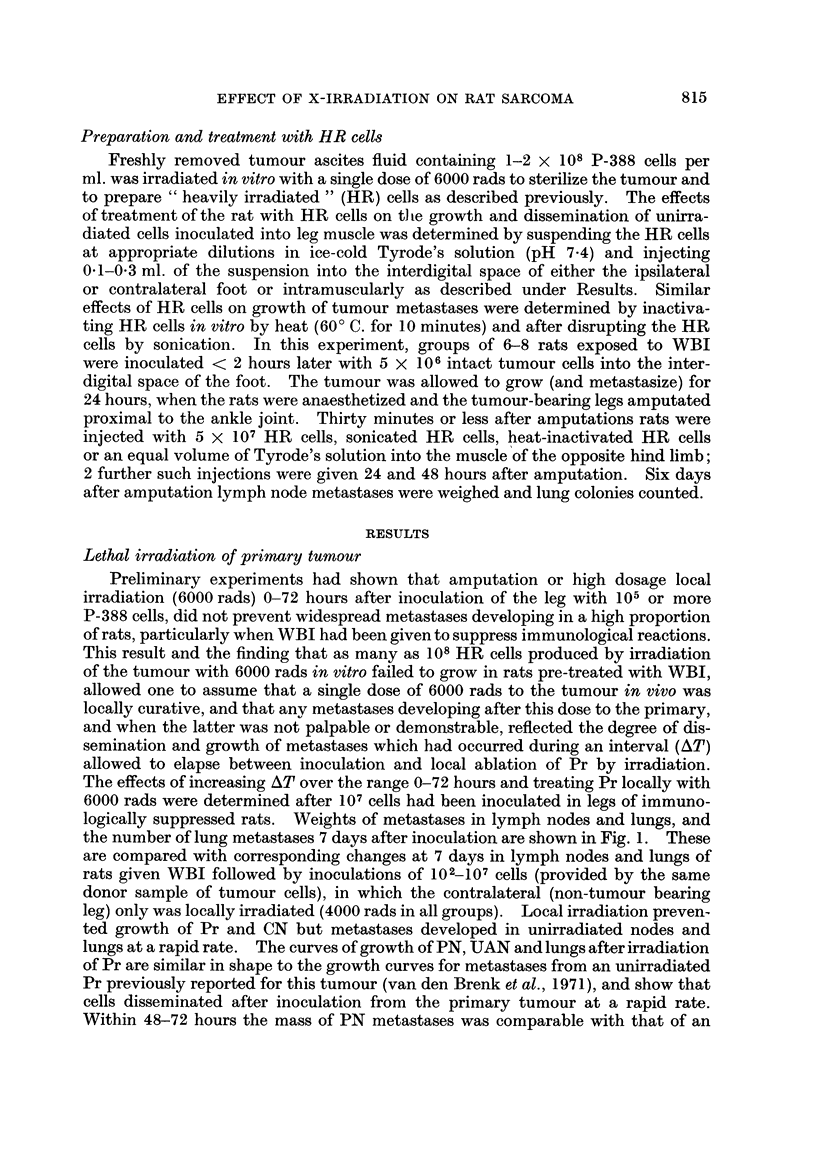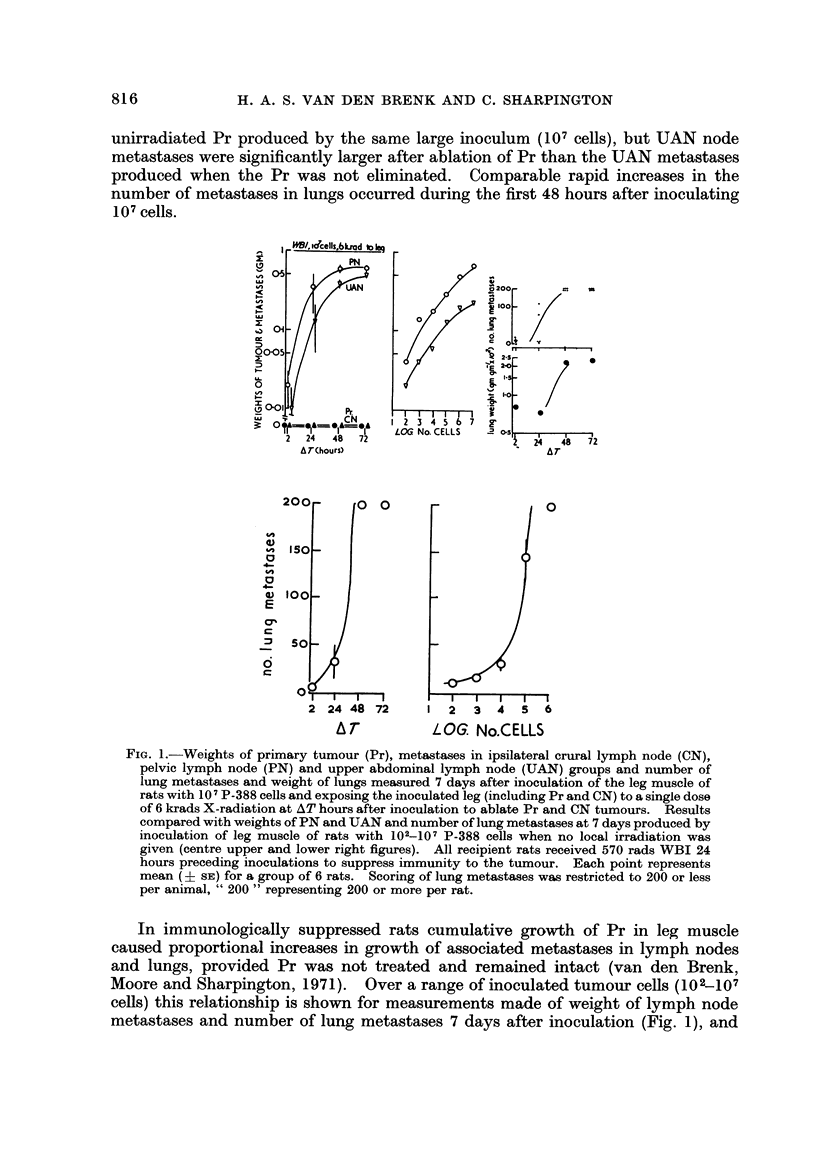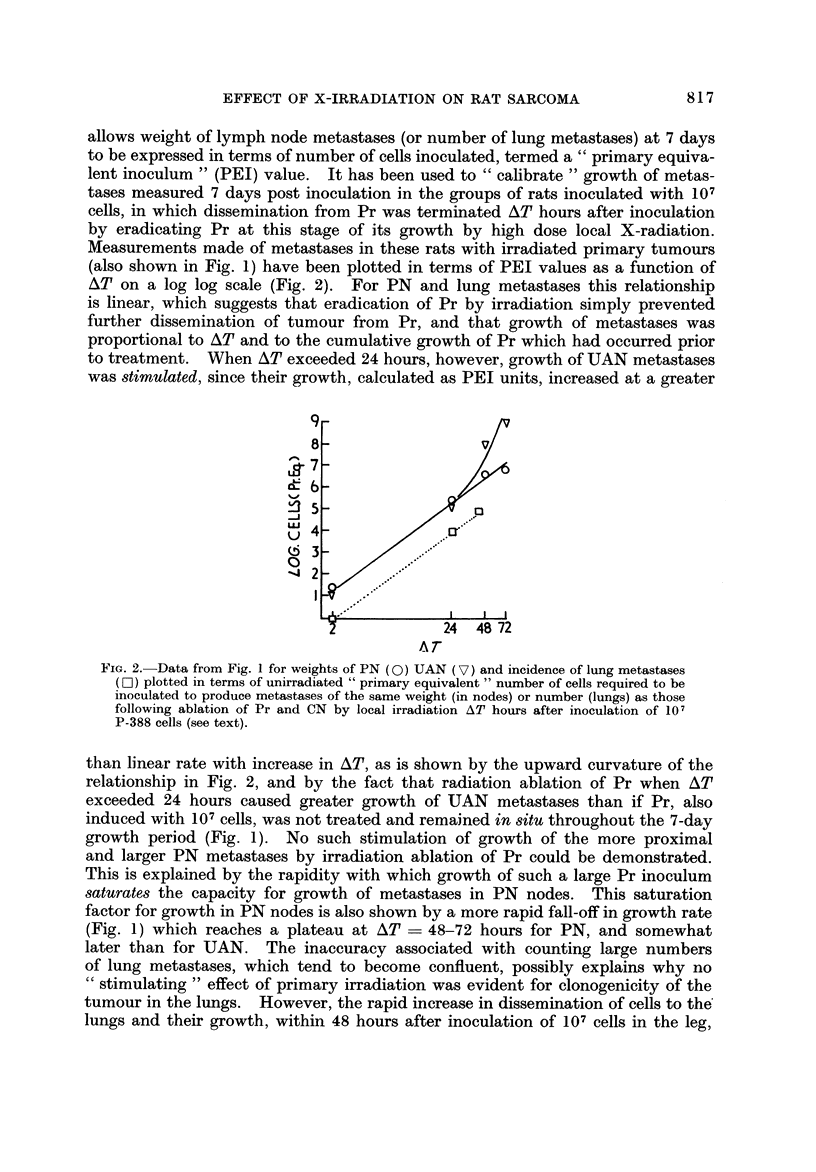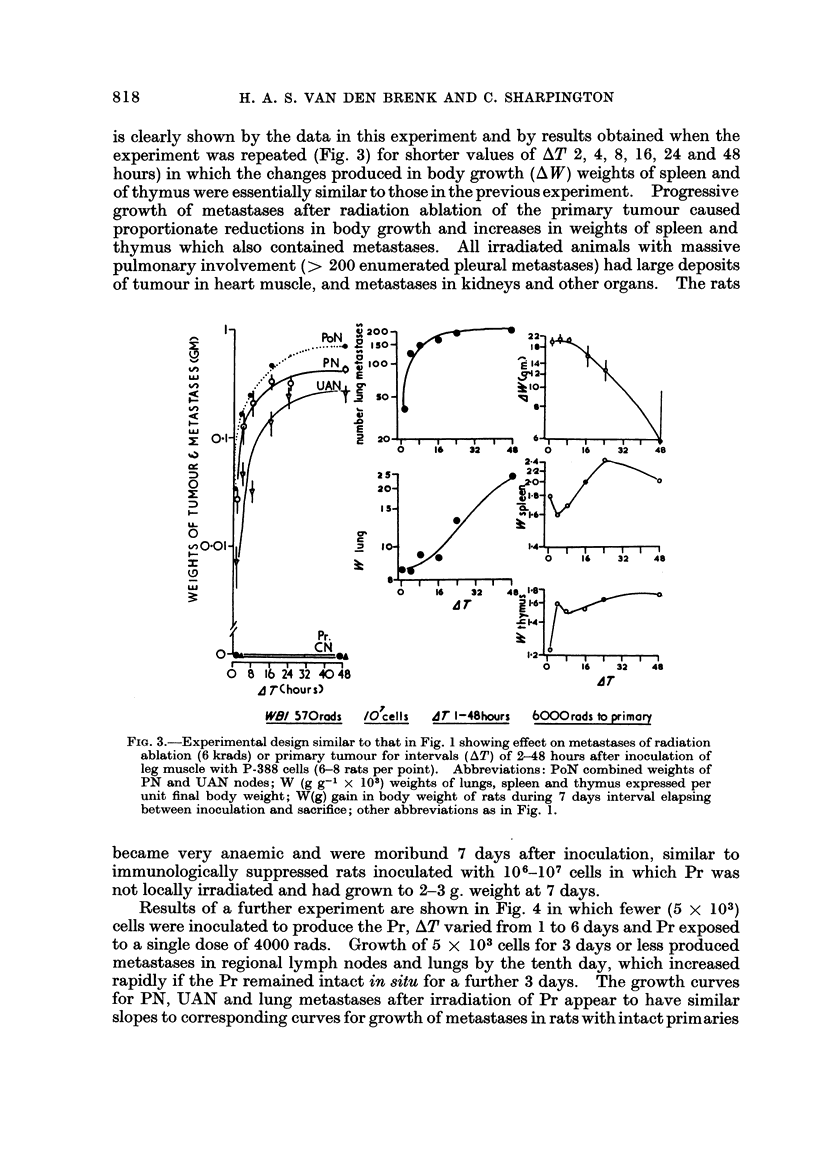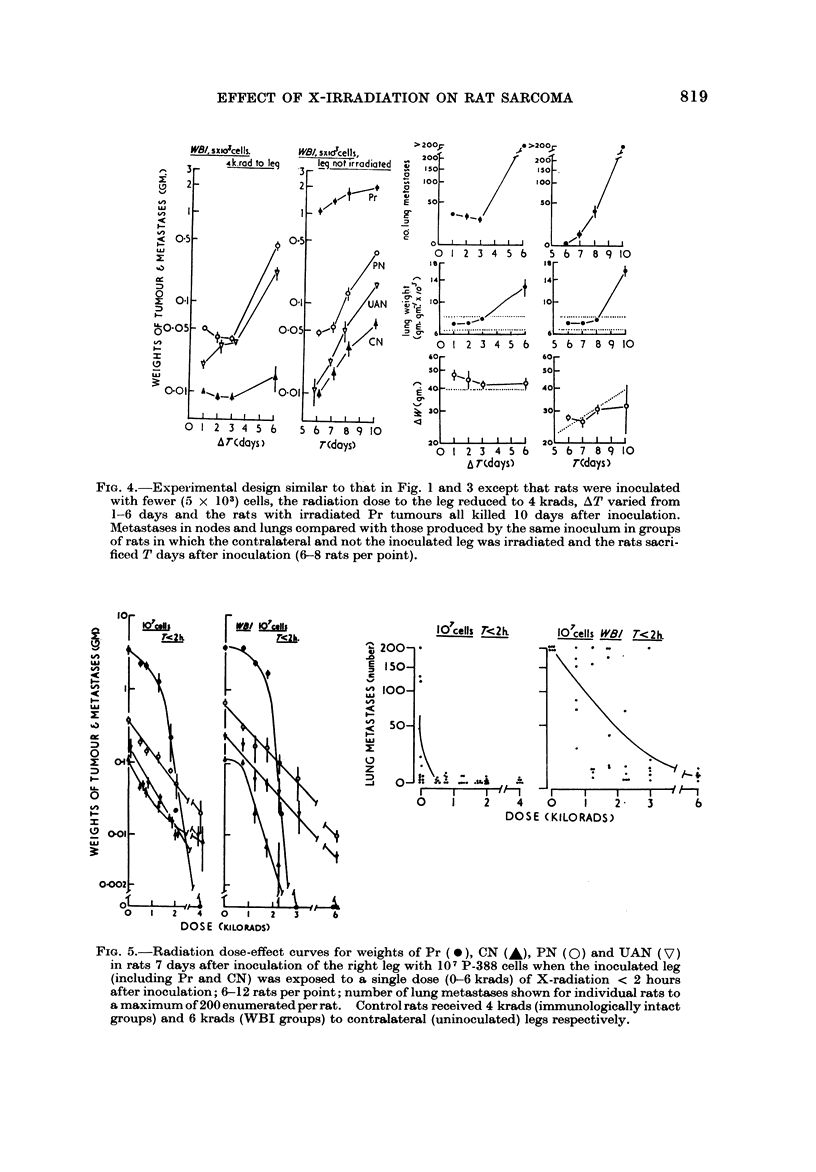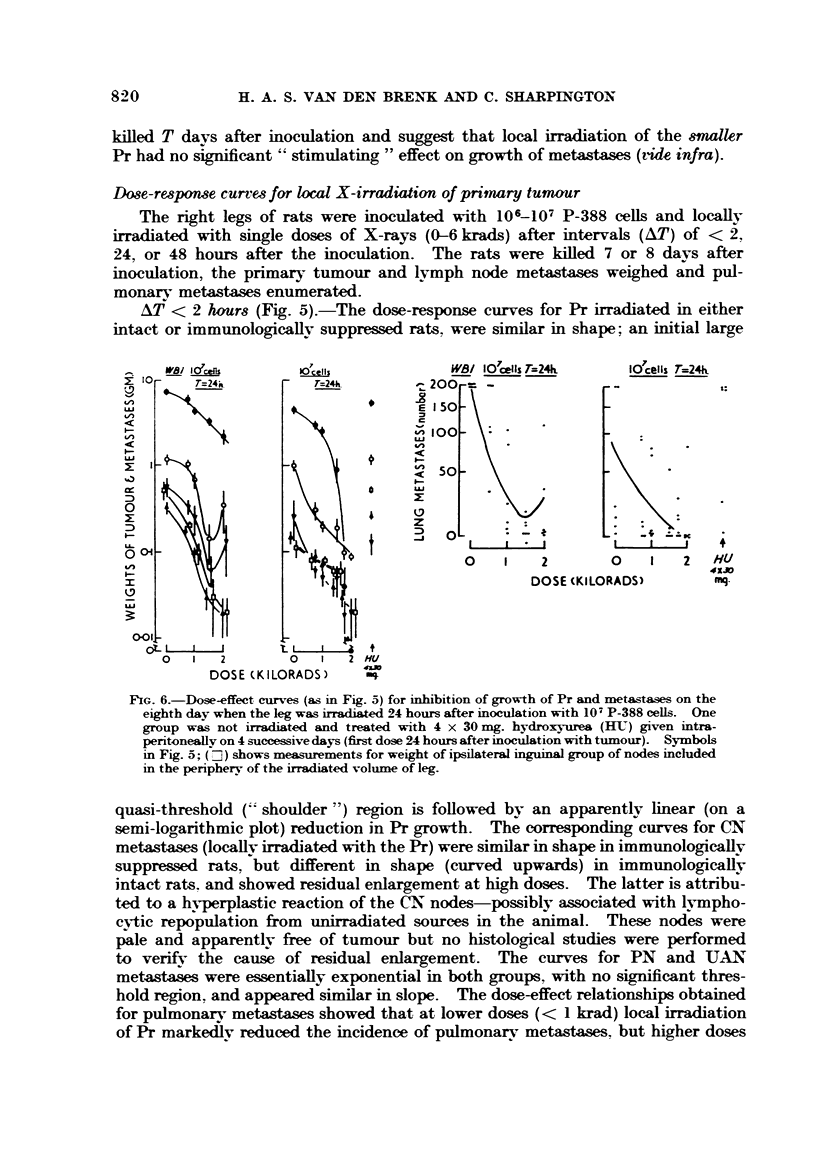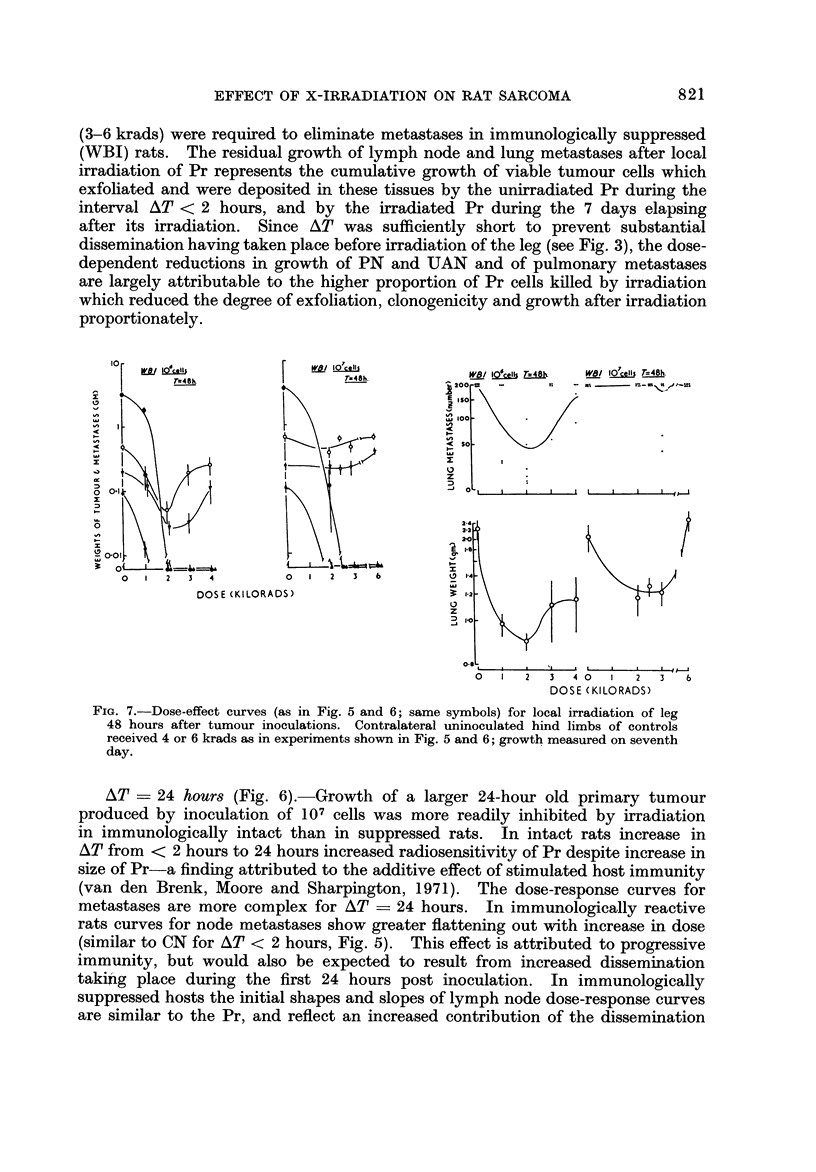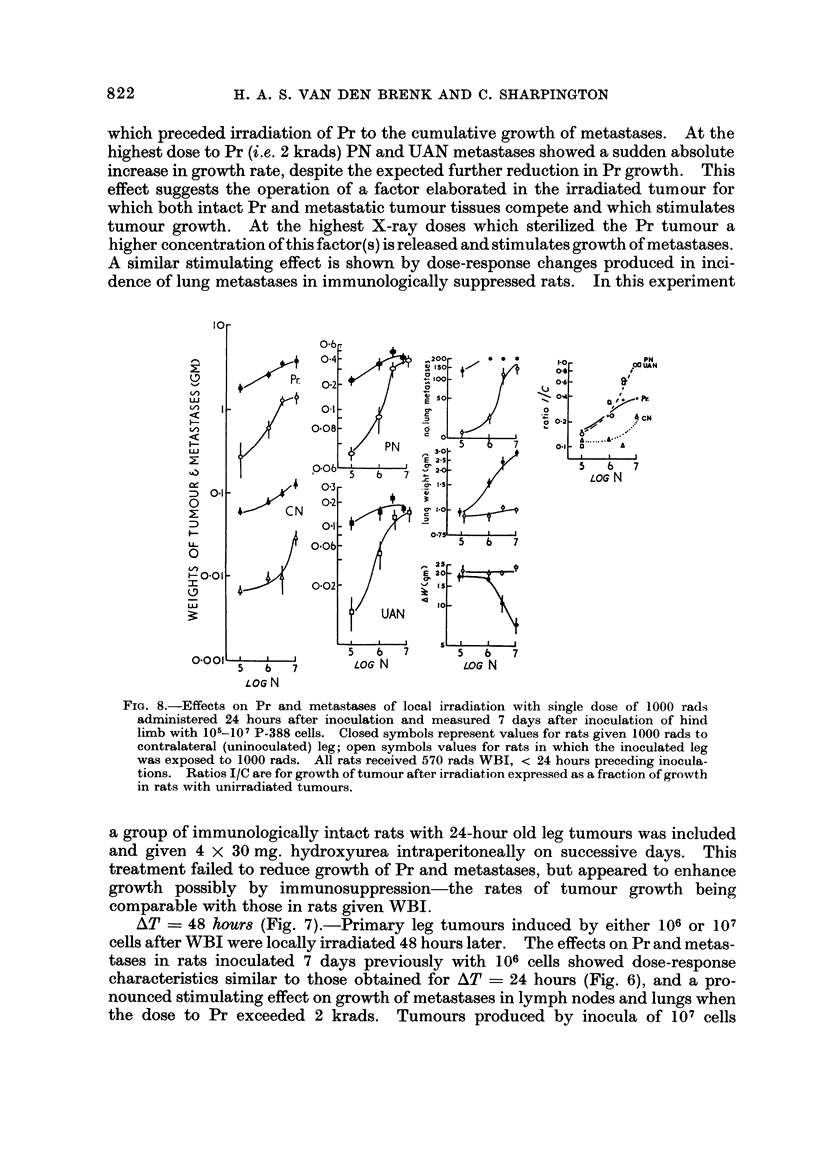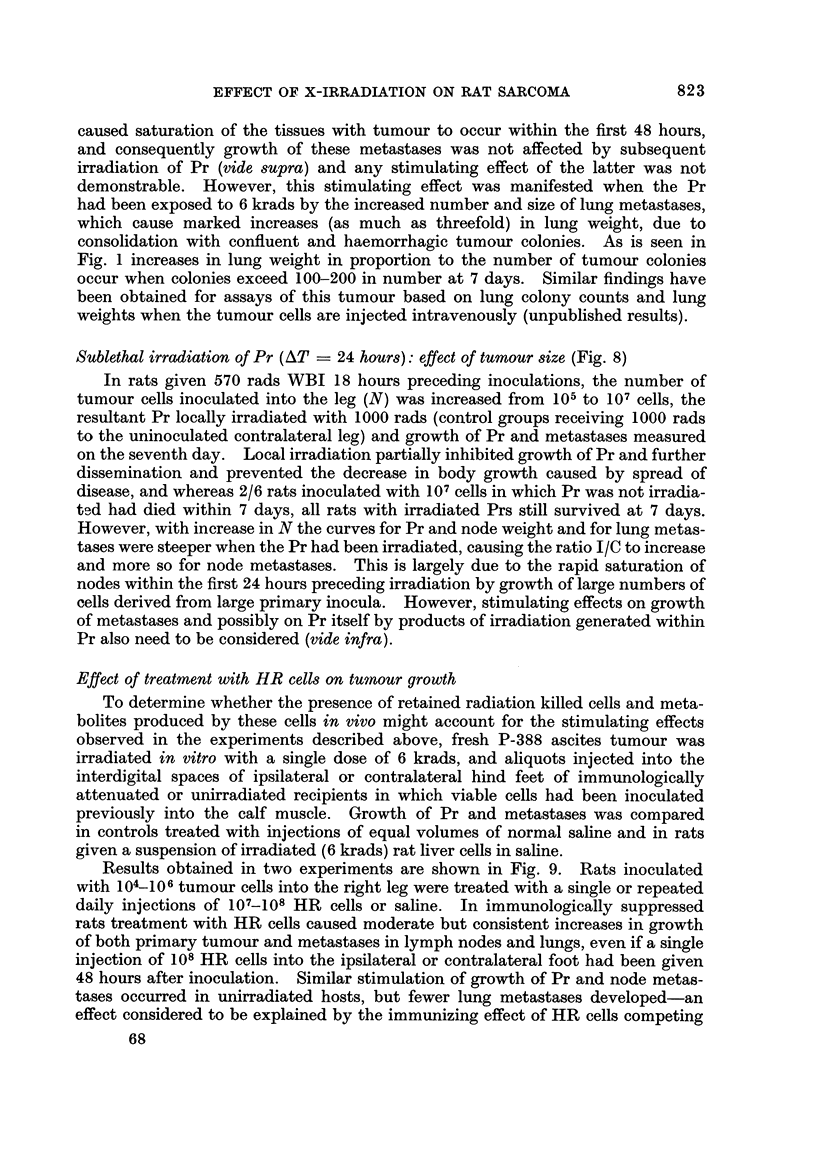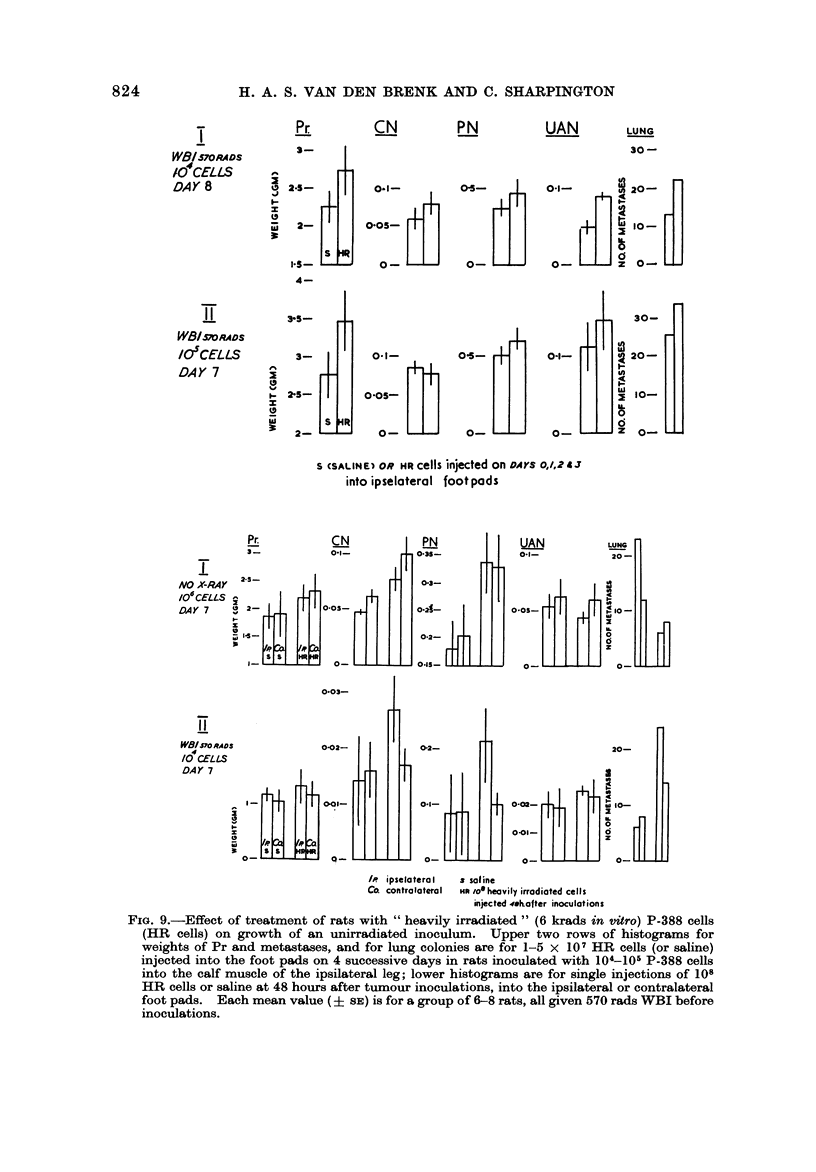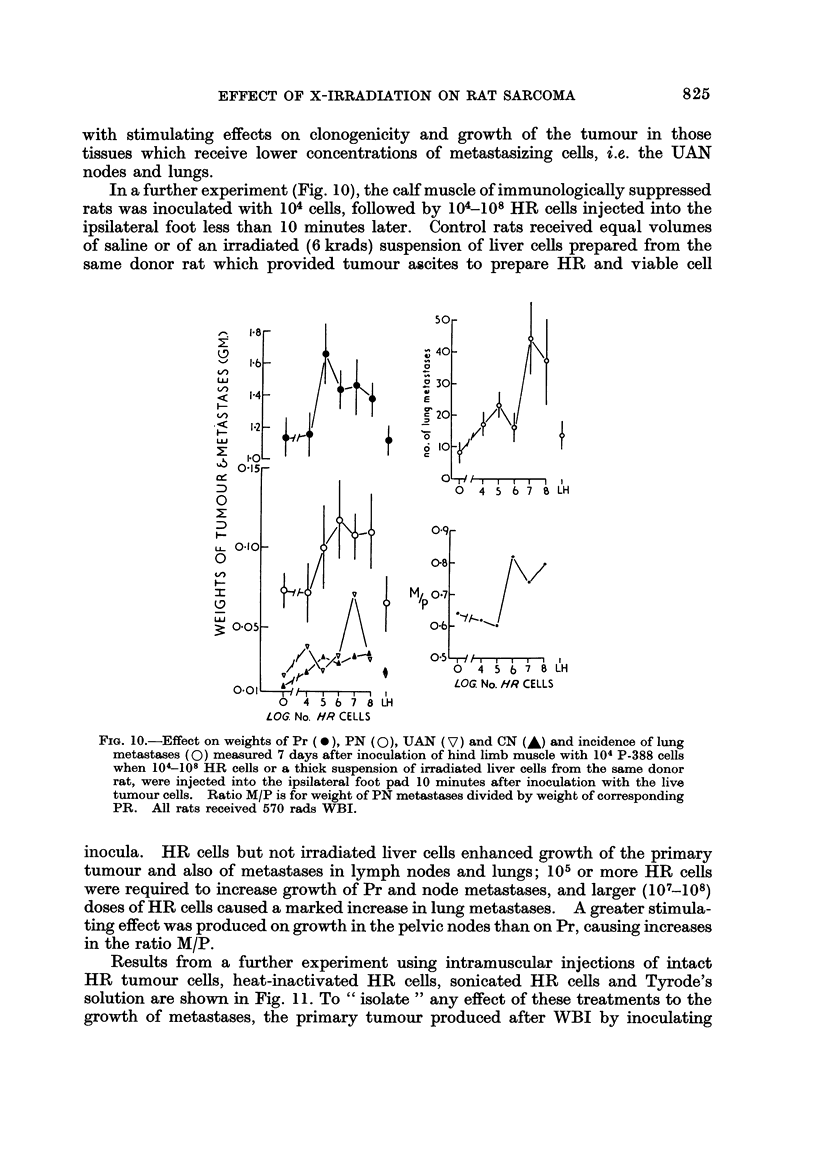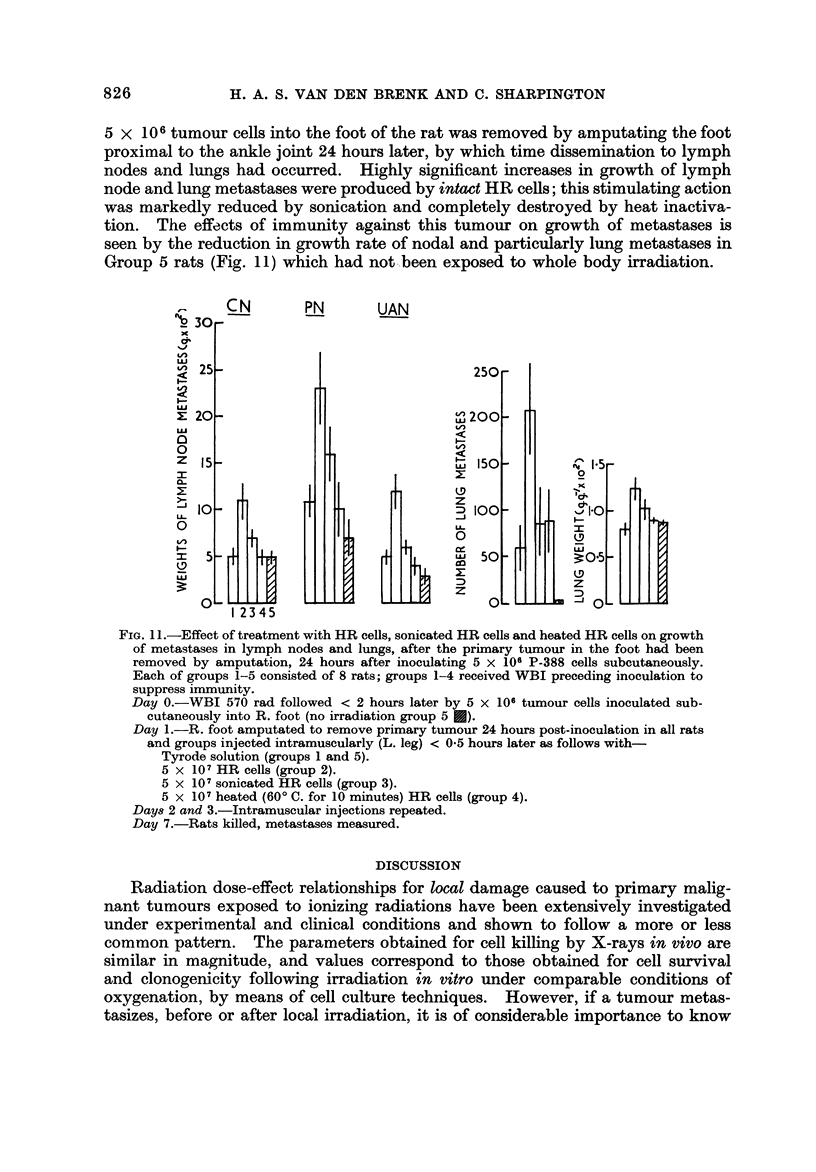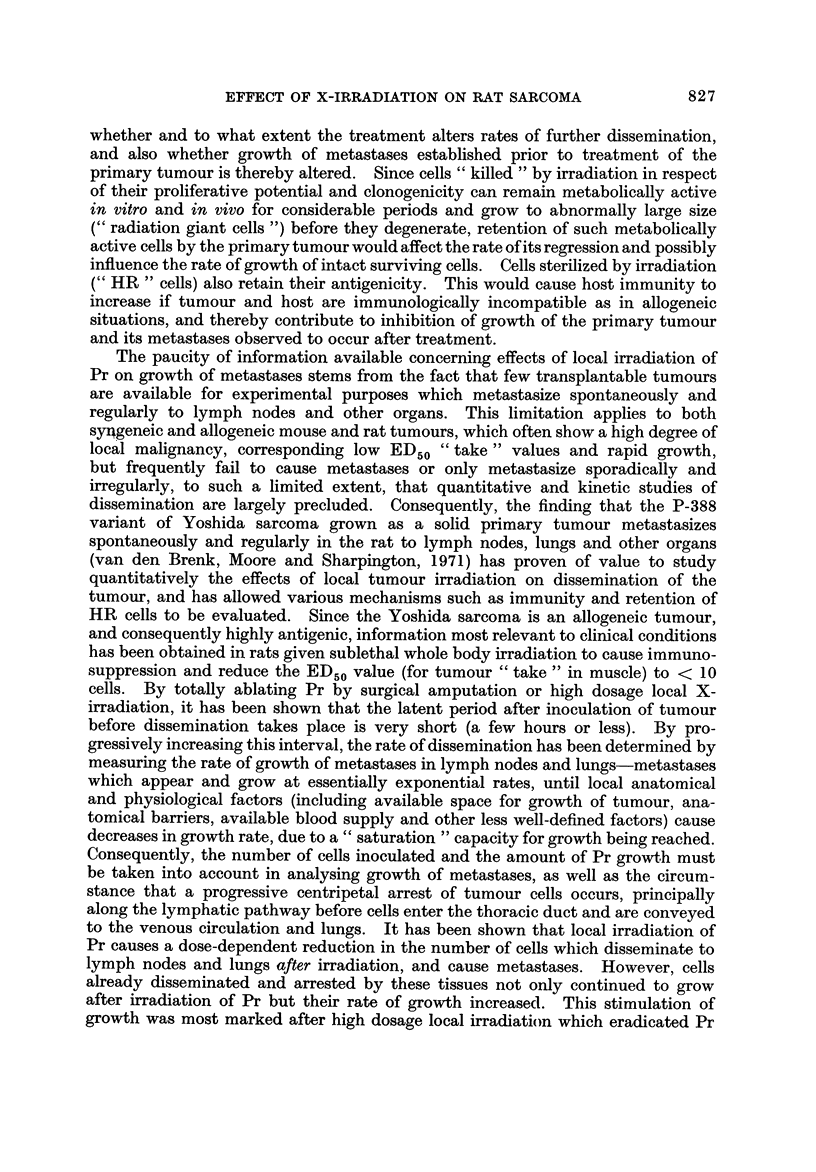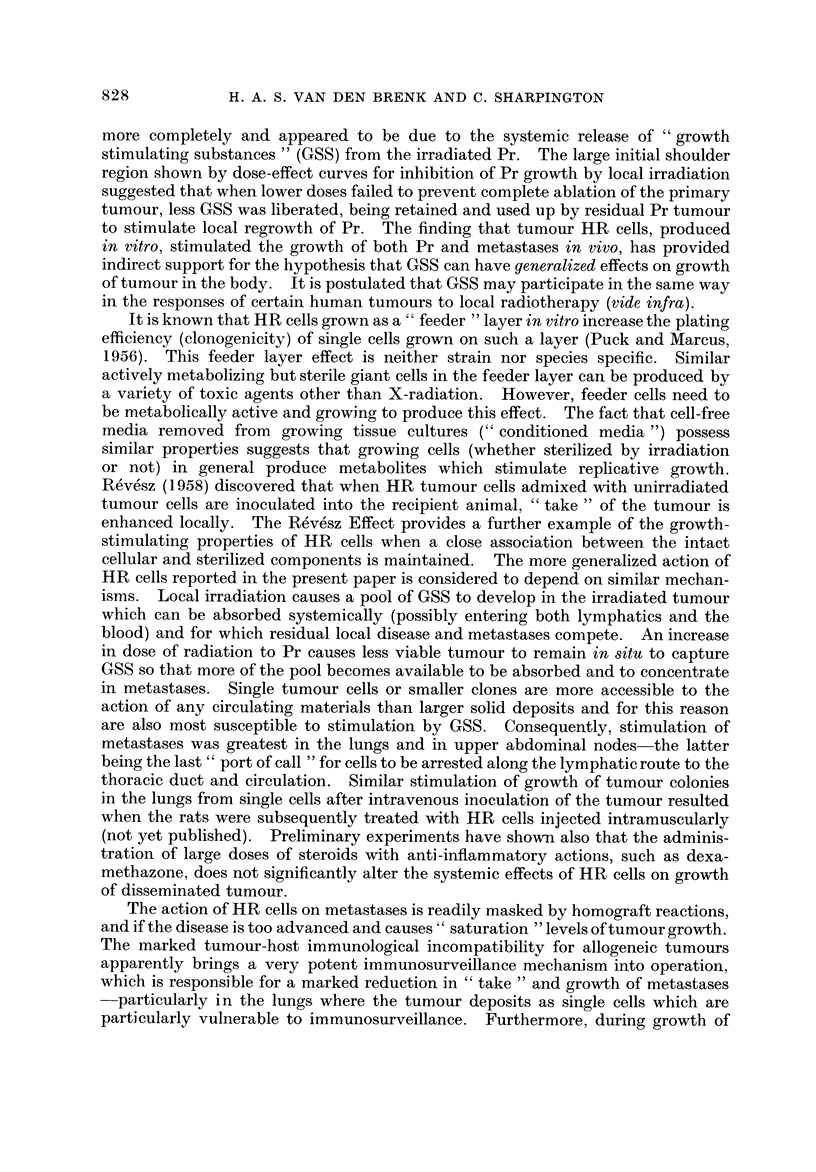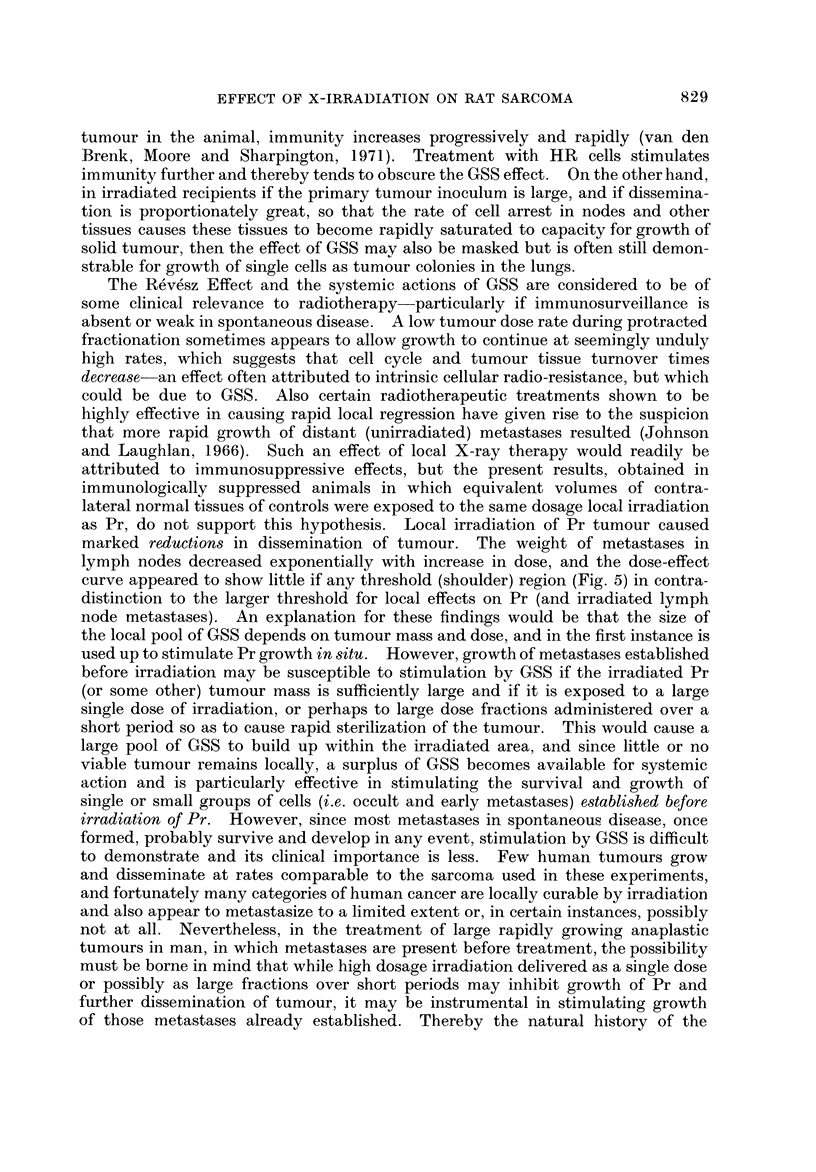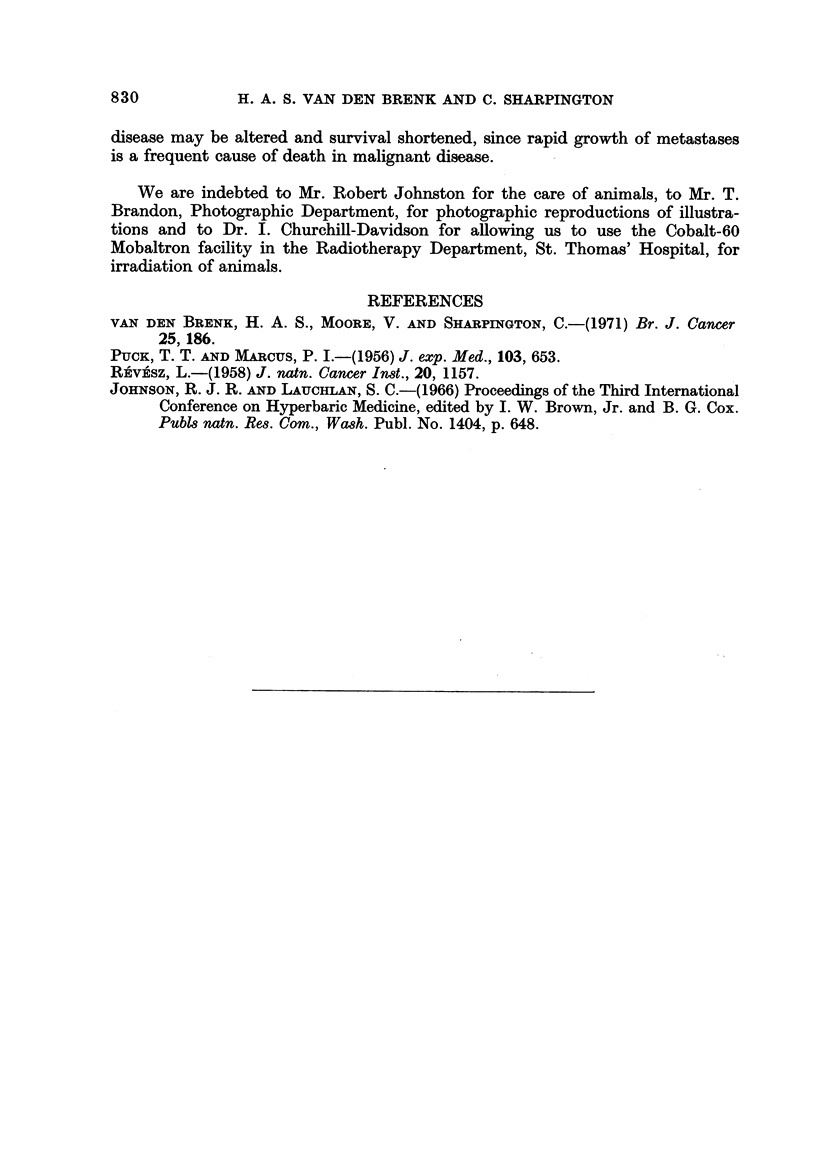# Effect of Local X-Irradiation of a Primary Sarcoma in the Rat on Dissemination and Growth of Metastases: Dose-Response Characteristics

**DOI:** 10.1038/bjc.1971.95

**Published:** 1971-12

**Authors:** H. A. S. van den Brenk, C. Sharpington

## Abstract

The effects of local X-irradiation of a solid, rapidly metastasizing sarcoma in the rat on kinetics of dissemination and growth of metastases in lymph nodes and lungs are described. Corresponding dose-effect curves obtained for growth of the primary tumour (Pr) and its metastases in unirradiated tissues showed that local irradiation of Pr caused an exponential decrease in growth of metastases due to any dissemination occurring after irradiation, but was also responsible for stimulating growth of metastases already established before treatment in lymph nodes and in lungs. This stimulating effect was most marked when Pr was larger at the time of treatment and when high doses were given to eradicate Pr. This effect is attributed to the liberation of growth stimulating substances (GSS) from a pool of GSS produced in the irradiated Pr by sterilized, but metabolically active and growing tumour cells (HR cells). This effect of HR cells on tumour growth and metastases was also demonstrated when rats were inoculated with viable tumour cells and subsequently treated by injecting large doses of HR cells prepared *in vitro,* into tissues remote from the Pr tumour site.

The systemic effects of GSS on metastases were most clearly seen after immunosuppression of recipient hosts by sublethal whole body irradiation, since immunosurveillance in unirradiated rats resulting from a rapidly developing allogenic tumour-host incompatibility caused marked reductions in clonogenicity of the tumour which tended to overshadow the GSS effect. The latter was also masked in immunosuppressed hosts when excessively high rates of dissemination were due to growth of large Pr inocula for sufficiently long to “saturate” the capacity for growth of metastatic tumour in lymph nodes and lungs.

The relevance of these findings to clinical radiotherapy is discussed.


### EFFECT OF LOCAL X-IRRADIATION OF A PRIMARY SARCOMA IN THE RAT ON DISSEMINATION AND GROWTH OF METASTASES: DOSE-RESPONSE CHARACTERISTICS

H. A. S. VAN DEN BRENK AND C. SHARPINGTON

*From the Richard Dimbleby Cancer Research Laboratory, St. Thomas' Hospital, London*

Received for publication October 14, 1971

**SUMMARY.—The effects of local X-irradiation of a solid, rapidly metastasizing sarcoma in the rat on kinetics of dissemination and growth of metastases in lymph nodes and lungs are described. Corresponding dose-effect curves obtained for growth of the primary tumour (Pr) and its metastases in unirradiated tissues showed that local irradiation of Pr caused an exponential decrease in growth of metastases due to any dissemination occurring after irradiation, but was also responsible for stimulating growth of metastases already established before treatment in lymph nodes and in lungs. This stimulating effect was most marked when Pr was larger at the time of treatment and when high doses were given to eradicate Pr. This effect is attributed to the liberation of growth stimulating substances (GSS) from a pool of GSS produced in the irradiated Pr by sterilized, but metabolically active and growing tumour cells (HR cells). This effect of HR cells on tumour growth and metastases was also demonstrated when rats were inoculated with viable tumour cells and subsequently treated by injecting large doses of HR cells prepared *in vitro*, into tissues remote from the Pr tumour site.**

**The systemic effects of GSS on metastases were most clearly seen after immunosuppression of recipient hosts by sublethal whole body irradiation, since immunosurveillance in unirradiated rats resulting from a rapidly developing allogenic tumour-host incompatibility caused marked reductions in clonogenicity of the tumour which tended to overshadow the GSS effect. The latter was also masked in immunosuppressed hosts when excessively high rates of dissemination were due to growth of large Pr inocula for sufficiently long to " saturate " the capacity for growth of metastatic tumour in lymph nodes and lungs.**

**The relevance of these findings to clinical radiotherapy is discussed.**

CONSIDERABLE information is available concerning the responses of primary tumours in animals and in man to local irradiation, but very few experimental studies have been carried out to determine the effects of local irradiation of a primary tumour on kinetics of dissemination and rates of growth of metastases. This is due to the fact that few transplantable solid tumours metastasize spontaneously with sufficient frequency and regularity for quantitative studies of tumour spread to be made. In a previous report (van den Brenk, Moore and Sharpington, 1971) kinetics of growth and dissemination of a rapidly growing transplantable allogeneic sarcoma in the rat have been described, and its metastatic behaviour in immunologically intact rats compared with that in immunologically suppressed and hyperimmune animals. The present paper describes radiation dose-response characteristics of growth of the transplanted primary tumour irradiated locally with single doses of X-rays *in vivo* and the effect it produced on dissemination and growth of metastases in lymph nodes and lungs. Since immunological (homograft) reactions profoundly affect rates of growth of this tumour (both primary and metastases) most observations and measurements have been made in rats exposed to sublethal whole body irradiation preceding inoculations with tumour, to suppress immunological reactions to its growth.

### MATERIALS AND METHODS

The mode of growth of the P-388 rat sarcoma, as an ascites or solid tumour, cell counting and transplantation techniques, and the specific pathogen free (SPF) strain of rats used in these experiments have been described previously (van den Brenk, Moore and Sharpington, 1971). Tumour cells inoculated into the leg muscle grow rapidly and produce a haemorrhagic solid tumour which disseminates along lymphatic pathways and causes solid metastases to develop, first in draining regional (pelvic and ipsilateral crural) lymph nodes, followed by centripetal spread to upper abdominal nodes. A high proportion of tumour cells which enter the thoracic duct and venous circulation are arrested in the lungs and proliferate to produce discrete clones (metastases) which grow to macroscopic size in 5–6 days. These can be counted on the pleural surfaces for quantitative studies. Consolidative growth of the pulmonary metastases also causes focal haemorrhage and oedema and increases in lung weight. The growth of pulmonary metastases causes rapid decline and death of animals. The number of tumour cells inoculated to produce palpable growth in leg muscle of 50% of recipients ($ED_{50}$) was $< 10$ cells in immunologically suppressed rats (pre-treated with 570 rads whole body irradiation), $\sim 5 \times 10^3$ cells in unirradiated (immunologically intact) rats and $> 10^5$ cells in hyperimmunized rats (*i.e.* rats pre-treated with heavily irradiated (HR) cells or in rats inoculated with viable tumour cells so as to produce a growing solid tumour before a second challenge of tumour cells was assayed in the contralateral leg muscle).

Only 5–7 week old female rats weighing 100–150 g. were used in the present study, and care was taken to select rats used in each experiment with body weights varying by not more than 10 g.

### *Local irradiation of leg tumours*

At various times ($<$ 1–72 hours) after inoculating the calf muscle of the right leg with the required number of tumour cells suspended in 0·1 ml. ice-cold Tyrode's solution (pH 7·4), the rat was anaesthetized with pentobarbital Na (36 mg. kg.$^{-1}$) or with methohexitone (33 mg. kg$^{-1}$) given intraperitoneally. Rats were irradiated on a specially constructed horizontal Perspex table which supported 2 parallel plates of lead, each 3 mm. thick and spaced 3 cm. apart. From each lead plate a 15 cm. diameter central window of lead had been removed. Each of 6 rats, arranged radially, was taped on to the lower plate of the lead sandwich so that only the leg to be irradiated distal to the inguinal ligament appeared within the frame of each opposed window, the remainder of the body of the rat being shielded by lead. The foot was taped down on to the Perspex platform, and further pieces of lead shielding were added to protect the foot distal to the ankle joint from irradiation.

The lead sandwich, mounted on the table, allowed the legs of 6 rats to be irradiated simultaneously and uniformly by inserting the assembly between 2 vertically mounted X-ray sources, operated at 250 kV and 15 mA with 1 mm. Cu plus 2 mm. Al added filtration (HVL 1 mm. Cu) to give a tissue dose rate measured in a simulated phantom with a Baldwin–Farmer secondary standard dosemeter of 304 rads $min^{-1}$. In the various experiments single doses to the leg within the range 100–6000 rads were administered. Control animals received the highest single dose used in a particular experiment to the uninoculated, contralateral (left) leg.

Since the popliteal region of the irradiated hind limb adjacent to the site of tumour cell inoculation was included in the irradiation field, any changes produced in weight of the ipsilateral popliteal (crural) nodes were due to the cumulative effects of growth of tumour cells deposited in the nodes before irradiation which survived, and the growth of intact cells disseminating to the nodes after irradiation from the primary tumour. Whole body irradiation causes atrophy of lymphoid tissues and prevents hyperplastic reactions to growth of this allogeneic tumour (van den Brenk, Moore and Sharpington, 1971). Consequently the weights of popliteal, pelvic and abdominal lymph nodes were reduced to $< 0{\cdot}01$ g., which was insignificant compared with the corresponding weights of metastases in nodes, and could be ignored in measuring weights of lymph node metastases.

*Whole body irradiation* (WBI)

A group of 10–12 rats placed in a ventilated Perspex box received 570 rads WBI at a dose rate of 125 rad $min^{-1}$ from the opposed beams of a $^{60}Co$ Mobaltron Unit (totalling 8273 Ci), 24 hours or less preceding tumour inoculations as described previously (van den Brenk, Moore and Sharpington, 1971).

*Measurement of tumour growth*

Most rats were sacrificed 7 days (some groups 6–10 days) *after inoculation* of the tumour. As described previously (van den Brenk, Moore and Sharpington, 1971), wet weights of the primary leg tumour (Pr), the ipsilateral (right) crural (or popliteal) nodes (CN), the pelvic or lower abdominal nodes (PN) and the coeliac group of upper abdominal nodes (UAN) were measured in each rat, together with weights of spleen, thymus and lungs. The total number of macroscopic metastases which could be recognized on the visceral pleural surfaces of the lungs were counted to a maximum of 200 per rat. Also recorded were macroscopic enlargement of inguinal, axillary and submandibular lymph nodes, the presence of tumour clones seen on the surfaces of the kidneys, and the macroscopic presence of lymphatic permeation along the superficial inguino-axillary vessels, which caused a pronounced local vasodilatation accompanied by tissue oedema and haemorrhagic staining of the peri-lymphatic tissues.

*Dose-effect curves*

Six to 8 rats were used at each radiation dose level given to the inoculated leg, and mean weights ($\pm$ SE) of Pr, CN, PN and UAN and incidence of lung metastases plotted as a function of dose received by Pr. Progressive increase in dimensions of Pr was determined by palpation and tumour size scored on a scale 0–6 units as described previously.

*Preparation and treatment with HR cells*

Freshly removed tumour ascites fluid containing 1–2 $\times$ $10^8$ P-388 cells per ml. was irradiated *in vitro* with a single dose of 6000 rads to sterilize the tumour and to prepare " heavily irradiated " (HR) cells as described previously. The effects of treatment of the rat with HR cells on the growth and dissemination of unirradiated cells inoculated into leg muscle was determined by suspending the HR cells at appropriate dilutions in ice-cold Tyrode's solution (pH 7·4) and injecting 0·1–0·3 ml. of the suspension into the interdigital space of either the ipsilateral or contralateral foot or intramuscularly as described under Results. Similar effects of HR cells on growth of tumour metastases were determined by inactivating HR cells *in vitro* by heat (60° C. for 10 minutes) and after disrupting the HR cells by sonication. In this experiment, groups of 6–8 rats exposed to WBI were inoculated $<$ 2 hours later with 5 $\times$ $10^6$ intact tumour cells into the interdigital space of the foot. The tumour was allowed to grow (and metastasize) for 24 hours, when the rats were anaesthetized and the tumour-bearing legs amputated proximal to the ankle joint. Thirty minutes or less after amputations rats were injected with 5 $\times$ $10^7$ HR cells, sonicated HR cells, heat-inactivated HR cells or an equal volume of Tyrode's solution into the muscle of the opposite hind limb; 2 further such injections were given 24 and 48 hours after amputation. Six days after amputation lymph node metastases were weighed and lung colonies counted.

### RESULTS

*Lethal irradiation of primary tumour*

Preliminary experiments had shown that amputation or high dosage local irradiation (6000 rads) 0–72 hours after inoculation of the leg with $10^5$ or more P-388 cells, did not prevent widespread metastases developing in a high proportion of rats, particularly when WBI had been given to suppress immunological reactions. This result and the finding that as many as $10^8$ HR cells produced by irradiation of the tumour with 6000 rads *in vitro* failed to grow in rats pre-treated with WBI, allowed one to assume that a single dose of 6000 rads to the tumour *in vivo* was locally curative, and that any metastases developing after this dose to the primary, and when the latter was not palpable or demonstrable, reflected the degree of dissemination and growth of metastases which had occurred during an interval ($\Delta T$) allowed to elapse between inoculation and local ablation of Pr by irradiation. The effects of increasing $\Delta T$ over the range 0–72 hours and treating Pr locally with 6000 rads were determined after $10^7$ cells had been inoculated in legs of immunologically suppressed rats. Weights of metastases in lymph nodes and lungs, and the number of lung metastases 7 days after inoculation are shown in Fig. 1. These are compared with corresponding changes at 7 days in lymph nodes and lungs of rats given WBI followed by inoculations of $10^2$–$10^7$ cells (provided by the same donor sample of tumour cells), in which the contralateral (non-tumour bearing leg) only was locally irradiated (4000 rads in all groups). Local irradiation prevented growth of Pr and CN but metastases developed in unirradiated nodes and lungs at a rapid rate. The curves of growth of PN, UAN and lungs after irradiation of Pr are similar in shape to the growth curves for metastases from an unirradiated Pr previously reported for this tumour (van den Brenk *et al.*, 1971), and show that cells disseminated after inoculation from the primary tumour at a rapid rate. Within 48–72 hours the mass of PN metastases was comparable with that of an unirradiated Pr produced by the same large inoculum ($10^7$ cells), but UAN node metastases were significantly larger after ablation of Pr than the UAN metastases produced when the Pr was not eliminated. Comparable rapid increases in the number of metastases in lungs occurred during the first 48 hours after inoculating $10^7$ cells.

Fig. 1.—Weights of primary tumour (Pr), metastases in ipsilateral crural lymph node (CN), pelvic lymph node (PN) and upper abdominal lymph node (UAN) groups and number of lung metastases and weight of lungs measured 7 days after inoculation of the leg muscle of rats with $10^7$ P-388 cells and exposing the inoculated leg (including Pr and CN) to a single dose of 6 krads X-radiation at $\Delta T$ hours after inoculation to ablate Pr and CN tumours. Results compared with weights of PN and UAN and number of lung metastases at 7 days produced by inoculation of leg muscle of rats with $10^2$–$10^7$ P-388 cells when no local irradiation was given (centre upper and lower right figures). All recipient rats received 570 rads WBI 24 hours preceding inoculations to suppress immunity to the tumour. Each point represents mean ($\pm$ SE) for a group of 6 rats. Scoring of lung metastases was restricted to 200 or less per animal, " 200 " representing 200 or more per rat.

In immunologically suppressed rats cumulative growth of Pr in leg muscle caused proportional increases in growth of associated metastases in lymph nodes and lungs, provided Pr was not treated and remained intact (van den Brenk, Moore and Sharpington, 1971). Over a range of inoculated tumour cells ($10^2$–$10^7$ cells) this relationship is shown for measurements made of weight of lymph node metastases and number of lung metastases 7 days after inoculation (Fig. 1), and allows weight of lymph node metastases (or number of lung metastases) at 7 days to be expressed in terms of number of cells inoculated, termed a " primary equivalent inoculum " (PEI) value. It has been used to " calibrate " growth of metastases measured 7 days post inoculation in the groups of rats inoculated with $10^7$ cells, in which dissemination from Pr was terminated $\Delta T$ hours after inoculation by eradicating Pr at this stage of its growth by high dose local X-radiation. Measurements made of metastases in these rats with irradiated primary tumours (also shown in Fig. 1) have been plotted in terms of PEI values as a function of $\Delta T$ on a log log scale (Fig. 2). For PN and lung metastases this relationship is linear, which suggests that eradication of Pr by irradiation simply prevented further dissemination of tumour from Pr, and that growth of metastases was proportional to $\Delta T$ and to the cumulative growth of Pr which had occurred prior to treatment. When $\Delta T$ exceeded 24 hours, however, growth of UAN metastases was *stimulated*, since their growth, calculated as PEI units, increased at a greater

FIG. 2.—Data from Fig. 1 for weights of PN (○) UAN (▽) and incidence of lung metastases (□) plotted in terms of unirradiated " primary equivalent " number of cells required to be inoculated to produce metastases of the same weight (in nodes) or number (lungs) as those following ablation of Pr and CN by local irradiation $\Delta T$ hours after inoculation of $10^7$ P-388 cells (see text).

than linear rate with increase in $\Delta T$, as is shown by the upward curvature of the relationship in Fig. 2, and by the fact that radiation ablation of Pr when $\Delta T$ exceeded 24 hours caused greater growth of UAN metastases than if Pr, also induced with $10^7$ cells, was not treated and remained *in situ* throughout the 7-day growth period (Fig. 1). No such stimulation of growth of the more proximal and larger PN metastases by irradiation ablation of Pr could be demonstrated. This is explained by the rapidity with which growth of such a large Pr inoculum *saturates* the capacity for growth of metastases in PN nodes. This saturation factor for growth in PN nodes is also shown by a more rapid fall-off in growth rate (Fig. 1) which reaches a plateau at $\Delta T$ = 48–72 hours for PN, and somewhat later than for UAN. The inaccuracy associated with counting large numbers of lung metastases, which tend to become confluent, possibly explains why no " stimulating " effect of primary irradiation was evident for clonogenicity of the tumour in the lungs. However, the rapid increase in dissemination of cells to the lungs and their growth, within 48 hours after inoculation of $10^7$ cells in the leg, is clearly shown by the data in this experiment and by results obtained when the experiment was repeated (Fig. 3) for shorter values of $\Delta T$ 2, 4, 8, 16, 24 and 48 hours) in which the changes produced in body growth ($\Delta W$) weights of spleen and of thymus were essentially similar to those in the previous experiment. Progressive growth of metastases after radiation ablation of the primary tumour caused proportionate reductions in body growth and increases in weights of spleen and thymus which also contained metastases. All irradiated animals with massive pulmonary involvement ($>$ 200 enumerated pleural metastases) had large deposits of tumour in heart muscle, and metastases in kidneys and other organs. The rats

FIG. 3.—Experimental design similar to that in Fig. 1 showing effect on metastases of radiation ablation (6 krads) or primary tumour for intervals ($\Delta T$) of 2–48 hours after inoculation of leg muscle with P-388 cells (6–8 rats per point). Abbreviations: PoN combined weights of PN and UAN nodes; W (g $g^{-1}$ $\times$ $10^3$) weights of lungs, spleen and thymus expressed per unit final body weight; W(g) gain in body weight of rats during 7 days interval elapsing between inoculation and sacrifice; other abbreviations as in Fig. 1.

became very anaemic and were moribund 7 days after inoculation, similar to immunologically suppressed rats inoculated with $10^6$–$10^7$ cells in which Pr was not locally irradiated and had grown to 2–3 g. weight at 7 days.

Results of a further experiment are shown in Fig. 4 in which fewer (5 $\times$ $10^3$) cells were inoculated to produce the Pr, $\Delta T$ varied from 1 to 6 days and Pr exposed to a single dose of 4000 rads. Growth of 5 $\times$ $10^3$ cells for 3 days or less produced metastases in regional lymph nodes and lungs by the tenth day, which increased rapidly if the Pr remained intact *in situ* for a further 3 days. The growth curves for PN, UAN and lung metastases after irradiation of Pr appear to have similar slopes to corresponding curves for growth of metastases in rats with intact primaries

FIG. 4.—Experimental design similar to that in Fig. 1 and 3 except that rats were inoculated with fewer ($5 \times 10^3$) cells, the radiation dose to the leg reduced to 4 krads, $\Delta T$ varied from 1–6 days and the rats with irradiated Pr tumours all killed 10 days after inoculation. Metastases in nodes and lungs compared with those produced by the same inoculum in groups of rats in which the contralateral and not the inoculated leg was irradiated and the rats sacrificed $T$ days after inoculation (6–8 rats per point).

FIG. 5.—Radiation dose-effect curves for weights of Pr (●), CN (▲), PN (○) and UAN (▽) in rats 7 days after inoculation of the right leg with $10^7$ P-388 cells when the inoculated leg (including Pr and CN) was exposed to a single dose (0–6 krads) of X-radiation < 2 hours after inoculation; 6–12 rats per point; number of lung metastases shown for individual rats to a maximum of 200 enumerated per rat. Control rats received 4 krads (immunologically intact groups) and 6 krads (WBI groups) to contralateral (uninoculated) legs respectively.

killed $T$ days after inoculation and suggest that local irradiation of the *smaller* Pr had no significant " stimulating " effect on growth of metastases (*vide infra*).

*Dose-response curves for local X-irradiation of primary tumour*

The right legs of rats were inoculated with $10^6$–$10^7$ P-388 cells and locally irradiated with single doses of X-rays (0–6 krads) after intervals ($\Delta T$) of $< 2$, 24, or 48 hours after the inoculation. The rats were killed 7 or 8 days after inoculation, the primary tumour and lymph node metastases weighed and pulmonary metastases enumerated.

$\Delta T < 2$ *hours* (Fig. 5).—The dose-response curves for Pr irradiated in either intact or immunologically suppressed rats, were similar in shape; an initial large

FIG. 6.—Dose-effect curves (as in Fig. 5) for inhibition of growth of Pr and metastases on the eighth day when the leg was irradiated 24 hours after inoculation with $10^7$ P-388 cells. One group was not irradiated and treated with 4 × 30 mg. hydroxyurea (HU) given intraperitoneally on 4 successive days (first dose 24 hours after inoculation with tumour). Symbols in Fig. 5; (□) shows measurements for weight of ipsilateral inguinal group of nodes included in the periphery of the irradiated volume of leg.

quasi-threshold (" shoulder ") region is followed by an apparently linear (on a semi-logarithmic plot) reduction in Pr growth. The corresponding curves for CN metastases (locally irradiated with the Pr) were similar in shape in immunologically suppressed rats, but different in shape (curved upwards) in immunologically intact rats, and showed residual enlargement at high doses. The latter is attributed to a hyperplastic reaction of the CN nodes—possibly associated with lymphocytic repopulation from unirradiated sources in the animal. These nodes were pale and apparently free of tumour but no histological studies were performed to verify the cause of residual enlargement. The curves for PN and UAN metastases were essentially exponential in both groups, with no significant threshold region, and appeared similar in slope. The dose-effect relationships obtained for pulmonary metastases showed that at lower doses ($< 1$ krad) local irradiation of Pr markedly reduced the incidence of pulmonary metastases, but higher doses

(3–6 krads) were required to eliminate metastases in immunologically suppressed (WBI) rats. The residual growth of lymph node and lung metastases after local irradiation of Pr represents the cumulative growth of viable tumour cells which exfoliated and were deposited in these tissues by the unirradiated Pr during the interval $\Delta T < 2$ hours, and by the irradiated Pr during the 7 days elapsing after its irradiation. Since $\Delta T$ was sufficiently short to prevent substantial dissemination having taken place before irradiation of the leg (see Fig. 3), the dose-dependent reductions in growth of PN and UAN and of pulmonary metastases are largely attributable to the higher proportion of Pr cells killed by irradiation which reduced the degree of exfoliation, clonogenicity and growth after irradiation proportionately.

FIG. 7.—Dose-effect curves (as in Fig. 5 and 6; same symbols) for local irradiation of leg 48 hours after tumour inoculations. Contralateral uninoculated hind limbs of controls received 4 or 6 krads as in experiments shown in Fig. 5 and 6; growth measured on seventh day.

$\Delta T = 24$ *hours* (Fig. 6).—Growth of a larger 24-hour old primary tumour produced by inoculation of $10^7$ cells was more readily inhibited by irradiation in immunologically intact than in suppressed rats. In intact rats increase in $\Delta T$ from $< 2$ hours to 24 hours increased radiosensitivity of Pr despite increase in size of Pr—a finding attributed to the additive effect of stimulated host immunity (van den Brenk, Moore and Sharpington, 1971). The dose-response curves for metastases are more complex for $\Delta T = 24$ hours. In immunologically reactive rats curves for node metastases show greater flattening out with increase in dose (similar to CN for $\Delta T < 2$ hours, Fig. 5). This effect is attributed to progressive immunity, but would also be expected to result from increased dissemination taking place during the first 24 hours post inoculation. In immunologically suppressed hosts the initial shapes and slopes of lymph node dose-response curves are similar to the Pr, and reflect an increased contribution of the dissemination which preceded irradiation of Pr to the cumulative growth of metastases. At the highest dose to Pr (*i.e.* 2 krads) PN and UAN metastases showed a sudden absolute increase in growth rate, despite the expected further reduction in Pr growth. This effect suggests the operation of a factor elaborated in the irradiated tumour for which both intact Pr and metastatic tumour tissues compete and which stimulates tumour growth. At the highest X-ray doses which sterilized the Pr tumour a higher concentration of this factor(s) is released and stimulates growth of metastases. A similar stimulating effect is shown by dose-response changes produced in incidence of lung metastases in immunologically suppressed rats. In this experiment

FIG. 8.—Effects on Pr and metastases of local irradiation with single dose of 1000 rads administered 24 hours after inoculation and measured 7 days after inoculation of hind limb with $10^5$–$10^7$ P-388 cells. Closed symbols represent values for rats given 1000 rads to contralateral (uninoculated) leg; open symbols values for rats in which the inoculated leg was exposed to 1000 rads. All rats received 570 rads WBI, < 24 hours preceding inoculations. Ratios I/C are for growth of tumour after irradiation expressed as a fraction of growth in rats with unirradiated tumours.

a group of immunologically intact rats with 24-hour old leg tumours was included and given 4 × 30 mg. hydroxyurea intraperitoneally on successive days. This treatment failed to reduce growth of Pr and metastases, but appeared to enhance growth possibly by immunosuppression—the rates of tumour growth being comparable with those in rats given WBI.

$\Delta T$ = 48 *hours* (Fig. 7).—Primary leg tumours induced by either $10^6$ or $10^7$ cells after WBI were locally irradiated 48 hours later. The effects on Pr and metastases in rats inoculated 7 days previously with $10^6$ cells showed dose-response characteristics similar to those obtained for $\Delta T$ = 24 hours (Fig. 6), and a pronounced stimulating effect on growth of metastases in lymph nodes and lungs when the dose to Pr exceeded 2 krads. Tumours produced by inocula of $10^7$ cells caused saturation of the tissues with tumour to occur within the first 48 hours, and consequently growth of these metastases was not affected by subsequent irradiation of Pr (*vide supra*) and any stimulating effect of the latter was not demonstrable. However, this stimulating effect was manifested when the Pr had been exposed to 6 krads by the increased number and size of lung metastases, which cause marked increases (as much as threefold) in lung weight, due to consolidation with confluent and haemorrhagic tumour colonies. As is seen in Fig. 1 increases in lung weight in proportion to the number of tumour colonies occur when colonies exceed 100–200 in number at 7 days. Similar findings have been obtained for assays of this tumour based on lung colony counts and lung weights when the tumour cells are injected intravenously (unpublished results).

*Sublethal irradiation of Pr* ($\Delta T = 24$ *hours*)*: effect of tumour size* (Fig. 8)

In rats given 570 rads WBI 18 hours preceding inoculations, the number of tumour cells inoculated into the leg (*N*) was increased from $10^5$ to $10^7$ cells, the resultant Pr locally irradiated with 1000 rads (control groups receiving 1000 rads to the uninoculated contralateral leg) and growth of Pr and metastases measured on the seventh day. Local irradiation partially inhibited growth of Pr and further dissemination and prevented the decrease in body growth caused by spread of disease, and whereas 2/6 rats inoculated with $10^7$ cells in which Pr was not irradiated had died within 7 days, all rats with irradiated Prs still survived at 7 days. However, with increase in *N* the curves for Pr and node weight and for lung metastases were steeper when the Pr had been irradiated, causing the ratio I/C to increase and more so for node metastases. This is largely due to the rapid saturation of nodes within the first 24 hours preceding irradiation by growth of large numbers of cells derived from large primary inocula. However, stimulating effects on growth of metastases and possibly on Pr itself by products of irradiation generated within Pr also need to be considered (*vide infra*).

*Effect of treatment with HR cells on tumour growth*

To determine whether the presence of retained radiation killed cells and metabolites produced by these cells *in vivo* might account for the stimulating effects observed in the experiments described above, fresh P-388 ascites tumour was irradiated *in vitro* with a single dose of 6 krads, and aliquots injected into the interdigital spaces of ipsilateral or contralateral hind feet of immunologically attenuated or unirradiated recipients in which viable cells had been inoculated previously into the calf muscle. Growth of Pr and metastases was compared in controls treated with injections of equal volumes of normal saline and in rats given a suspension of irradiated (6 krads) rat liver cells in saline.

Results obtained in two experiments are shown in Fig. 9. Rats inoculated with $10^4$–$10^6$ tumour cells into the right leg were treated with a single or repeated daily injections of $10^7$–$10^8$ HR cells or saline. In immunologically suppressed rats treatment with HR cells caused moderate but consistent increases in growth of both primary tumour and metastases in lymph nodes and lungs, even if a single injection of $10^8$ HR cells into the ipsilateral or contralateral foot had been given 48 hours after inoculation. Similar stimulation of growth of Pr and node metastases occurred in unirradiated hosts, but fewer lung metastases developed—an effect considered to be explained by the immunizing effect of HR cells competing

Fig. 9.—Effect of treatment of rats with " heavily irradiated " (6 krads *in vitro*) P-388 cells (HR cells) on growth of an unirradiated inoculum. Upper two rows of histograms for weights of Pr and metastases, and for lung colonies are for 1–5 $\times$ $10^7$ HR cells (or saline) injected into the foot pads on 4 successive days in rats inoculated with $10^4$–$10^5$ P-388 cells into the calf muscle of the ipsilateral leg; lower histograms are for single injections of $10^8$ HR cells or saline at 48 hours after tumour inoculations, into the ipsilateral or contralateral foot pads. Each mean value ($\pm$ SE) is for a group of 6–8 rats, all given 570 rads WBI before inoculations.

with stimulating effects on clonogenicity and growth of the tumour in those tissues which receive lower concentrations of metastasizing cells, *i.e.* the UAN nodes and lungs.

In a further experiment (Fig. 10), the calf muscle of immunologically suppressed rats was inoculated with $10^4$ cells, followed by $10^4$–$10^8$ HR cells injected into the ipsilateral foot less than 10 minutes later. Control rats received equal volumes of saline or of an irradiated (6 krads) suspension of liver cells prepared from the same donor rat which provided tumour ascites to prepare HR and viable cell

FIG. 10.—Effect on weights of Pr (●), PN (○), UAN (▽) and CN (▲) and incidence of lung metastases (○) measured 7 days after inoculation of hind limb muscle with $10^4$ P-388 cells when $10^4$–$10^8$ HR cells or a thick suspension of irradiated liver cells from the same donor rat, were injected into the ipsilateral foot pad 10 minutes after inoculation with the live tumour cells. Ratio M/P is for weight of PN metastases divided by weight of corresponding PR. All rats received 570 rads WBI.

inocula. HR cells but not irradiated liver cells enhanced growth of the primary tumour and also of metastases in lymph nodes and lungs; $10^5$ or more HR cells were required to increase growth of Pr and node metastases, and larger ($10^7$–$10^8$) doses of HR cells caused a marked increase in lung metastases. A greater stimulating effect was produced on growth in the pelvic nodes than on Pr, causing increases in the ratio M/P.

Results from a further experiment using intramuscular injections of intact HR tumour cells, heat-inactivated HR cells, sonicated HR cells and Tyrode's solution are shown in Fig. 11. To " isolate " any effect of these treatments to the growth of metastases, the primary tumour produced after WBI by inoculating

$5 \times 10^6$ tumour cells into the foot of the rat was removed by amputating the foot proximal to the ankle joint 24 hours later, by which time dissemination to lymph nodes and lungs had occurred. Highly significant increases in growth of lymph node and lung metastases were produced by *intact* HR cells; this stimulating action was markedly reduced by sonication and completely destroyed by heat inactivation. The effects of immunity against this tumour on growth of metastases is seen by the reduction in growth rate of nodal and particularly lung metastases in Group 5 rats (Fig. 11) which had not been exposed to whole body irradiation.

FIG. 11.—Effect of treatment with HR cells, sonicated HR cells and heated HR cells on growth of metastases in lymph nodes and lungs, after the primary tumour in the foot had been removed by amputation, 24 hours after inoculating $5 \times 10^6$ P-388 cells subcutaneously. Each of groups 1–5 consisted of 8 rats; groups 1–4 received WBI preceding inoculation to suppress immunity.

*Day* 0.—WBI 570 rad followed < 2 hours later by $5 \times 10^6$ tumour cells inoculated subcutaneously into R. foot (no irradiation group 5 ▨).

*Day* 1.—R. foot amputated to remove primary tumour 24 hours post-inoculation in all rats and groups injected intramuscularly (L. leg) < 0·5 hours later as follows with—

Tyrode solution (groups 1 and 5).

$5 \times 10^7$ HR cells (group 2).

$5 \times 10^7$ sonicated HR cells (group 3).

$5 \times 10^7$ heated (60° C. for 10 minutes) HR cells (group 4).

*Days* 2 *and* 3.—Intramuscular injections repeated.

*Day* 7.—Rats killed, metastases measured.

### DISCUSSION

Radiation dose-effect relationships for *local* damage caused to primary malignant tumours exposed to ionizing radiations have been extensively investigated under experimental and clinical conditions and shown to follow a more or less common pattern. The parameters obtained for cell killing by X-rays *in vivo* are similar in magnitude, and values correspond to those obtained for cell survival and clonogenicity following irradiation *in vitro* under comparable conditions of oxygenation, by means of cell culture techniques. However, if a tumour metastasizes, before or after local irradiation, it is of considerable importance to know whether and to what extent the treatment alters rates of further dissemination, and also whether growth of metastases established prior to treatment of the primary tumour is thereby altered. Since cells " killed " by irradiation in respect of their proliferative potential and clonogenicity can remain metabolically active *in vitro* and *in vivo* for considerable periods and grow to abnormally large size (" radiation giant cells ") before they degenerate, retention of such metabolically active cells by the primary tumour would affect the rate of its regression and possibly influence the rate of growth of intact surviving cells. Cells sterilized by irradiation (" HR " cells) also retain their antigenicity. This would cause host immunity to increase if tumour and host are immunologically incompatible as in allogeneic situations, and thereby contribute to inhibition of growth of the primary tumour and its metastases observed to occur after treatment.

The paucity of information available concerning effects of local irradiation of Pr on growth of metastases stems from the fact that few transplantable tumours are available for experimental purposes which metastasize spontaneously and regularly to lymph nodes and other organs. This limitation applies to both syngeneic and allogeneic mouse and rat tumours, which often show a high degree of local malignancy, corresponding low $ED_{50}$ " take " values and rapid growth, but frequently fail to cause metastases or only metastasize sporadically and irregularly, to such a limited extent, that quantitative and kinetic studies of dissemination are largely precluded. Consequently, the finding that the P-388 variant of Yoshida sarcoma grown as a solid primary tumour metastasizes spontaneously and regularly in the rat to lymph nodes, lungs and other organs (van den Brenk, Moore and Sharpington, 1971) has proven of value to study quantitatively the effects of local tumour irradiation on dissemination of the tumour, and has allowed various mechanisms such as immunity and retention of HR cells to be evaluated. Since the Yoshida sarcoma is an allogeneic tumour, and consequently highly antigenic, information most relevant to clinical conditions has been obtained in rats given sublethal whole body irradiation to cause immuno-suppression and reduce the $ED_{50}$ value (for tumour " take " in muscle) to $< 10$ cells. By totally ablating Pr by surgical amputation or high dosage local X-irradiation, it has been shown that the latent period after inoculation of tumour before dissemination takes place is very short (a few hours or less). By progressively increasing this interval, the rate of dissemination has been determined by measuring the rate of growth of metastases in lymph nodes and lungs—metastases which appear and grow at essentially exponential rates, until local anatomical and physiological factors (including available space for growth of tumour, anatomical barriers, available blood supply and other less well-defined factors) cause decreases in growth rate, due to a " saturation " capacity for growth being reached. Consequently, the number of cells inoculated and the amount of Pr growth must be taken into account in analysing growth of metastases, as well as the circumstance that a progressive centripetal arrest of tumour cells occurs, principally along the lymphatic pathway before cells enter the thoracic duct and are conveyed to the venous circulation and lungs. It has been shown that local irradiation of Pr causes a dose-dependent reduction in the number of cells which disseminate to lymph nodes and lungs *after* irradiation, and cause metastases. However, cells already disseminated and arrested by these tissues not only continued to grow after irradiation of Pr but their rate of growth increased. This stimulation of growth was most marked after high dosage local irradiation which eradicated Pr more completely and appeared to be due to the systemic release of " growth stimulating substances " (GSS) from the irradiated Pr. The large initial shoulder region shown by dose-effect curves for inhibition of Pr growth by local irradiation suggested that when lower doses failed to prevent complete ablation of the primary tumour, less GSS was liberated, being retained and used up by residual Pr tumour to stimulate local regrowth of Pr. The finding that tumour HR cells, produced *in vitro*, stimulated the growth of both Pr and metastases *in vivo*, has provided indirect support for the hypothesis that GSS can have *generalized* effects on growth of tumour in the body. It is postulated that GSS may participate in the same way in the responses of certain human tumours to local radiotherapy (*vide infra*).

It is known that HR cells grown as a " feeder " layer *in vitro* increase the plating efficiency (clonogenicity) of single cells grown on such a layer (Puck and Marcus, 1956). This feeder layer effect is neither strain nor species specific. Similar actively metabolizing but sterile giant cells in the feeder layer can be produced by a variety of toxic agents other than X-radiation. However, feeder cells need to be metabolically active and growing to produce this effect. The fact that cell-free media removed from growing tissue cultures (" conditioned media ") possess similar properties suggests that growing cells (whether sterilized by irradiation or not) in general produce metabolites which stimulate replicative growth. Révész (1958) discovered that when HR tumour cells admixed with unirradiated tumour cells are inoculated into the recipient animal, " take " of the tumour is enhanced locally. The Révész Effect provides a further example of the growth-stimulating properties of HR cells when a close association between the intact cellular and sterilized components is maintained. The more generalized action of HR cells reported in the present paper is considered to depend on similar mechanisms. Local irradiation causes a pool of GSS to develop in the irradiated tumour which can be absorbed systemically (possibly entering both lymphatics and the blood) and for which residual local disease and metastases compete. An increase in dose of radiation to Pr causes less viable tumour to remain *in situ* to capture GSS so that more of the pool becomes available to be absorbed and to concentrate in metastases. Single tumour cells or smaller clones are more accessible to the action of any circulating materials than larger solid deposits and for this reason are also most susceptible to stimulation by GSS. Consequently, stimulation of metastases was greatest in the lungs and in upper abdominal nodes—the latter being the last " port of call " for cells to be arrested along the lymphatic route to the thoracic duct and circulation. Similar stimulation of growth of tumour colonies in the lungs from single cells after intravenous inoculation of the tumour resulted when the rats were subsequently treated with HR cells injected intramuscularly (not yet published). Preliminary experiments have shown also that the administration of large doses of steroids with anti-inflammatory actions, such as dexamethazone, does not significantly alter the systemic effects of HR cells on growth of disseminated tumour.

The action of HR cells on metastases is readily masked by homograft reactions, and if the disease is too advanced and causes " saturation " levels of tumour growth. The marked tumour-host immunological incompatibility for allogeneic tumours apparently brings a very potent immunosurveillance mechanism into operation, which is responsible for a marked reduction in " take " and growth of metastases —particularly in the lungs where the tumour deposits as single cells which are particularly vulnerable to immunosurveillance. Furthermore, during growth of tumour in the animal, immunity increases progressively and rapidly (van den Brenk, Moore and Sharpington, 1971). Treatment with HR cells stimulates immunity further and thereby tends to obscure the GSS effect. On the other hand, in irradiated recipients if the primary tumour inoculum is large, and if dissemination is proportionately great, so that the rate of cell arrest in nodes and other tissues causes these tissues to become rapidly saturated to capacity for growth of solid tumour, then the effect of GSS may also be masked but is often still demonstrable for growth of single cells as tumour colonies in the lungs.

The Révész Effect and the systemic actions of GSS are considered to be of some clinical relevance to radiotherapy—particularly if immunosurveillance is absent or weak in spontaneous disease. A low tumour dose rate during protracted fractionation sometimes appears to allow growth to continue at seemingly unduly high rates, which suggests that cell cycle and tumour tissue turnover times *decrease*—an effect often attributed to intrinsic cellular radio-resistance, but which could be due to GSS. Also certain radiotherapeutic treatments shown to be highly effective in causing rapid local regression have given rise to the suspicion that more rapid growth of distant (unirradiated) metastases resulted (Johnson and Laughlan, 1966). Such an effect of local X-ray therapy would readily be attributed to immunosuppressive effects, but the present results, obtained in immunologically suppressed animals in which equivalent volumes of contralateral normal tissues of controls were exposed to the same dosage local irradiation as Pr, do not support this hypothesis. Local irradiation of Pr tumour caused marked *reductions* in dissemination of tumour. The weight of metastases in lymph nodes decreased exponentially with increase in dose, and the dose-effect curve appeared to show little if any threshold (shoulder) region (Fig. 5) in contradistinction to the larger threshold for local effects on Pr (and irradiated lymph node metastases). An explanation for these findings would be that the size of the local pool of GSS depends on tumour mass and dose, and in the first instance is used up to stimulate Pr growth *in situ*. However, growth of metastases established before irradiation may be susceptible to stimulation by GSS if the irradiated Pr (or some other) tumour mass is sufficiently large and if it is exposed to a large single dose of irradiation, or perhaps to large dose fractions administered over a short period so as to cause rapid sterilization of the tumour. This would cause a large pool of GSS to build up within the irradiated area, and since little or no viable tumour remains locally, a surplus of GSS becomes available for systemic action and is particularly effective in stimulating the survival and growth of single or small groups of cells (*i.e.* occult and early metastases) *established before irradiation of Pr*. However, since most metastases in spontaneous disease, once formed, probably survive and develop in any event, stimulation by GSS is difficult to demonstrate and its clinical importance is less. Few human tumours grow and disseminate at rates comparable to the sarcoma used in these experiments, and fortunately many categories of human cancer are locally curable by irradiation and also appear to metastasize to a limited extent or, in certain instances, possibly not at all. Nevertheless, in the treatment of large rapidly growing anaplastic tumours in man, in which metastases are present before treatment, the possibility must be borne in mind that while high dosage irradiation delivered as a single dose or possibly as large fractions over short periods may inhibit growth of Pr and further dissemination of tumour, it may be instrumental in stimulating growth of those metastases already established. Thereby the natural history of the disease may be altered and survival shortened, since rapid growth of metastases is a frequent cause of death in malignant disease.

We are indebted to Mr. Robert Johnston for the care of animals, to Mr. T. Brandon, Photographic Department, for photographic reproductions of illustrations and to Dr. I. Churchill-Davidson for allowing us to use the Cobalt-60 Mobaltron facility in the Radiotherapy Department, St. Thomas' Hospital, for irradiation of animals.

### REFERENCES

VAN DEN BRENK, H. A. S., MOORE, V. AND SHARPINGTON, C.—(1971) *Br. J. Cancer* **25**, **186**.

PUCK, T. T. AND MARCUS, P. I.—(1956) *J. exp. Med.*, **103**, 653.

RÉVÉSZ, L.—(1958) *J. natn. Cancer Inst.*, **20**, 1157.

JOHNSON, R. J. R. AND LAUCHLAN, S. C.—(1966) Proceedings of the Third International Conference on Hyperbaric Medicine, edited by I. W. Brown, Jr. and B. G. Cox. *Publs natn. Res. Com., Wash.* Publ. No. 1404, p. 648.